# Accuracy of Transcutaneous Carbon Dioxide Measurement During Transcatheter Aortic Valve Replacement Under Monitored Anesthesia Care: A Prospective Observational Study

**DOI:** 10.7759/cureus.53661

**Published:** 2024-02-05

**Authors:** Yuki Okazawa, Yuki Kataoka, Kazuo Shindo

**Affiliations:** 1 Department of Pharmacoepidemiology, Kyoto University Graduate School of Medicine/School of Public Health, Kyoto, JPN; 2 Department of Anesthesia, Hyogo Prefectural Amagasaki General Medical Center, Amagasaki, JPN; 3 Section of Clinical Epidemiology, Department of Community Medicine, Kyoto University Graduate School of Medicine, Kyoto, JPN; 4 Department of Healthcare Epidemiology, Kyoto University Graduate School of Medicine/School of Public Health, Kyoto, JPN; 5 Department of Systematic Reviewers, Scientific Research Works Peer Support Group, Osaka, JPN; 6 Department of Internal Medicine, Kyoto Min-iren Asukai Hospital, Kyoto, JPN

**Keywords:** non-invasive monitoring, procedural sedation and analgesia, transcatheter aortic valve repair, transcutaneous carbon dioxide tension, monitored anesthesia care (mac)

## Abstract

Background

Transcutaneous carbon dioxide tension (PtcCO_2_) measurement is a promising alternative to arterial carbon dioxide tension (PaCO_2_) measurement. PaCO_2_ measurement is invasive and intermittent, whereas PtcCO_2_ measurement is non-invasive and continuous. However, previous studies evaluating PtcCO_2_measurements did not include patients undergoing transcatheter aortic valve replacement (TAVR), who experience anticipated hemodynamic changes, particularly before and after valve placement. Therefore, we investigated whether PtcCO_2_ measurement could provide an alternative to PaCO_2_ measurement during transfemoral TAVR under monitored anesthesia care (MAC) with local anesthesia.

Methodology

We conducted a prospective observational study. We included all consecutive patients with severe aortic stenosis who were scheduled to undergo a transfemoral TAVR under MAC at our institution from November 1, 2020, to April 30, 2021. During the procedures, PaCO_2_ and PtcCO_2_ were concurrently monitored six times as a reference standard and index test, respectively. PtcCO_2_ was monitored continuously using a non-invasive earlobe sensor. The agreement between PtcCO_2_ and PaCO_2_ measurements was assessed using the Bland-Altman method, and the 95% limits of agreement were calculated. Based on previous studies, we determined that 95% limits of agreement of ±6.0 mmHg would be clinically acceptable to define PtcCO_2_ as an alternative to PaCO_2_.

Results

We obtained 88 measurement pairs from 15 patients. The lower and upper 95% limits of agreement between the PtcCO_2_ and PaCO_2_ measurements were -4.22 mmHg and 6.56 mmHg, respectively.

Conclusions

During TAVR under MAC with local anesthesia, PtcCO_2_ measurement could not provide a viable alternative to PaCO_2_ measurement to reduce high PaCO2 events. This study focused on comparing intraoperative periods before and after valve implantation. Therefore, further investigation is warranted to assess the impact of various factors, including the prosthetic valve type and the hemodynamic effects of balloon aortic valvuloplasty, on PtcCO_2_ measurement in TAVR.

## Introduction

Transcatheter aortic valve replacement (TAVR) has become a revolutionary treatment for elderly patients with severe symptomatic aortic valve stenosis [1]. Initially, general anesthesia (GA) was the principal anesthetic technique used during TAVR. However, as clinicians gained experience and devices improved, transfemoral TAVR is increasingly being performed under monitored anesthesia care (MAC) or procedural sedation and analgesia instead of GA [2,3]. Furthermore, registry studies have reported lower 30-day mortality rates with procedural sedation and analgesia than with GA [2,3], and a randomized clinical trial reported that vasopressors or inotropes were used less frequently during conscious sedation than during GA [4].

When TAVR is performed under MAC rather than GA, careful respiratory status monitoring is essential. The risk of hypercapnia and respiratory acidosis is significantly higher for patients undergoing transfemoral TAVR under sedation than it is for those undergoing the procedure under GA [5]. Moreover, respiratory distress, which may arise from factors such as pulmonary congestion due to the supine position, aspiration, or overdose of sedatives, has been attributed as the cause of emergency conversion from sedation to GA during TAVR in 23% of patients [6]. In addition to these respiratory effects, the hemodynamic effects of hypercapnia must also be considered. In a study of healthy young adult male volunteers, an increase in arterial carbon dioxide tension (PaCO_2_) from 38.7 (0.3) mmHg (mean (standard error)) to 50.2 (0.9) mmHg caused a corresponding increase in left ventricular workload, arterial pressure, and heart rate. This is thought to be due to sympathetic nervous system activation [7]. This sympathetic stimulation may lead to adverse cardiovascular events in patients with severe aortic stenosis (AS). In patients with AS, there is already a state of elevated left ventricular pressure. Thus, hypercapnic respiratory acidosis is a serious potential complication during TAVR under MAC. To prevent respiratory distress, it is necessary to monitor changes in respiratory rate and consciousness level, as well as pay attention to changes in PaCO_2_.

PaCO_2_ is measured by arterial blood gas analysis. Other methods to assess PaCO_2_ include measurement of end-tidal carbon dioxide tension (PetCO_2_) and transcutaneous carbon dioxide tension (PtcCO_2_). PetCO_2_, measured by capnography, is sometimes used to monitor respiratory patterns during sedation. This is useful because the waveform of the capnography can be used to monitor the patient’s respiratory pattern [8]. However, the PetCO_2_ value itself does not always accurately reflect PaCO_2_. Several studies have addressed the differences between PaCO_2_ and PetCO_2_ measured by capnography [9,10]. The patient’s breathing pattern, such as apnea or hypopnea, affects PetCO_2_ measurement. This leads to unreliable absolute values of PetCO_2_. PetCO_2_ underestimates PaCO_2_ in spontaneously breathing patients [9,10].

By contrast, PtcCO_2_ measurement has been reported as a promising alternative to PaCO_2_ measurement in recent years. PaCO_2_ measurement offers intermittent data, while PtcCO_2_ measurement is non-invasive and enables continuous monitoring. PtcCO_2_ measurement can be performed by attaching a sensor to the skin, and good compatibility with PaCO_2_ has been reported [11-16]. However, previous studies evaluating PtcCO_2_ measurements did not include patients undergoing TAVR, who experience anticipated hemodynamic changes, particularly before and after valve placement [17-19].

Therefore, this study investigated whether PtcCO_2_ measurement could provide an alternative to PaCO_2_ measurement during transfemoral TAVR under MAC with local anesthesia.

As a side note, we presented this study’s results at the 26th Annual Meeting of the Japanese Society of Cardiovascular Anesthesiologists in Kanazawa, Japan, on October 14, 2021.

## Materials and methods

Reporting guidelines and approvals

This study followed the STAndards for Reporting Diagnostic accuracy studies (i.e., STARD) 2015 reporting guidelines [20]. Furthermore, our single-center, prospective, observational study protocol was approved by the Institutional Review Board of Hyogo Prefectural Amagasaki General Medical Center, Hyogo, Japan (#2-135; approved on October 20, 2020). We did not register the protocol on a publicly accessible server, but the protocol was filed with the Institutional Review Board. All participants (or their next of kin) provided written informed consent before entering the study and could withdraw consent at any time.

Participants

This study included all consecutive patients with severe AS who were scheduled for transfemoral TAVR under MAC with local anesthesia at our institution from November 1, 2020, to April 30, 2021.

Patients who preoperatively used vasoactive drugs, required intraoperative conversion to GA, had earlobe skin disorders, or did not provide consent were excluded.

Monitored anesthesia care with local anesthesia strategies

MAC was achieved by continuous dexmedetomidine infusion and an intermittent bolus of propofol (0.1-0.2 mg/kg) or midazolam (0.02 mg/kg). The dexmedetomidine infusion was started at a rate of 1.4 µg/kg/hour for approximately 10 minutes until there was no response to the call and was reduced to 0.7 µg/kg/hour. Analgesia was provided by intermittent intravenous fentanyl (0.5-2 µg/kg), and local anesthesia was administered at the puncture site. The attending anesthesiologists titrated the sedative and analgesic drug doses to induce moderate-to-deep sedation [21] and maintain adequate spontaneous ventilation during the TAVR procedure. Particularly during the placement of the prosthetic valve, the sedation level was adjusted to achieve deep sedation. The choice of sedative and analgesic techniques was at the discretion of the anesthesiologists. Interventionalists applied local anesthesia (1% lidocaine) at the puncture site and adjusted the local anesthesia dosage.

Study protocol and measurements

We monitored PaCO_2_ as a reference standard and PtcCO_2_ as an index test. PaCO_2_ was measured with the ABL800 FLEX® blood gas analyzer (Radiometer, Copenhagen, Denmark) and PtcCO_2_ was monitored continuously using a non-invasive TCM5® earlobe sensor (Radiometer, Copenhagen, Denmark) heated to 42°C. Automatic calibration using the integrated system in TCM5® was conducted for each patient before device placement. The electrode was attached to the earlobe skin with a clip. To facilitate contact with the electrode, we swabbed the earlobe with alcohol before placing the electrode. We also monitored the patients with a five-lead electrocardiogram and measured oxygen saturation by pulse oximetry, non-invasive blood pressure, invasive arterial blood pressure, and the bispectral index.

We performed six simultaneous PtcCO_2_ and PaCO_2_ measurements during the TAVR procedure. It has been reported that PtcCO_2_ values tend to lag several minutes behind PaCO_2_ measurements in their response [22]. Therefore, measurements were taken at points where the hemodynamic and respiratory statuses were relatively stable. The PtcCO_2_ and PaCO_2_ results were available to the attending anesthesiologists for clinical decision-making. The first (T1), second (T2), and third (T3) measurements were taken before valve implantation and the fourth (T4), fifth (T5), and sixth (T6) measurements were taken after valve implantation. T1 was measured immediately after insertion of the radial arterial catheter; T2 and T3 were measured two and thirty minutes after the first heparin administration, respectively. T4 was measured five minutes after the implantation of the prosthetic valve, T5 was measured two minutes after the first protamine administration, and T6 was measured at the end of surgery.

We also collected baseline data for each participant including age, sex, the Society of Thoracic Surgeons mortality score [23], and the presence of comorbidities. Additionally, we recorded intraoperative data, including anesthesia and procedure times, type of valve used in the procedure, use of sedatives, analgesics, or vasopressors, and their dosages.

Statistical analysis

The patient characteristics and intraoperative data were presented as the mean (standard deviation, SD) or absolute values with percentages, as appropriate.

We analyzed the agreement between the measurements of PtcCO_2_ and PaCO_2_ by Bland-Altman analysis and calculated 95% limits of agreement [24]. A study of surgical intensive care unit (ICU) patients established a clinically acceptable range of 7.5 mmHg as clinically acceptable to define the two methods as interchangeable [11]. In addition, other studies comparing the measurements of PtcCO_2_ and PaCO_2_ [25,26] were referenced, and we determined that 95% limits of agreement of ±6.0 mmHg would be clinically acceptable to define PtcCO_2 _as an alternative to PaCO_2_. Applying a two-sided type 1 error rate of 5% with 80% power, 83 pairs of measurements were calculated to be required to detect 95% limits of agreement with a mean difference of 1.0 mmHg and an SD of 2.0 mmHg [27]. We planned to take six intraoperative measurements per patient. Therefore, 14 patients were required. We decided to monitor 20 patients to allow for possible missing values. All analyses were performed using RStudio Cloud Version 1.4.1718-1 (The R Foundation for Statistical Computing, Vienna, Austria).

Post hoc analysis

Recognizing the possibility of fixed and proportional errors between the mean and the difference between the two measurements, we performed a post hoc linear univariate regression analysis using “mean of measures” as the independent variable and “difference of measures” as the dependent variable.

## Results

Participant demographics

**Figure 1:**
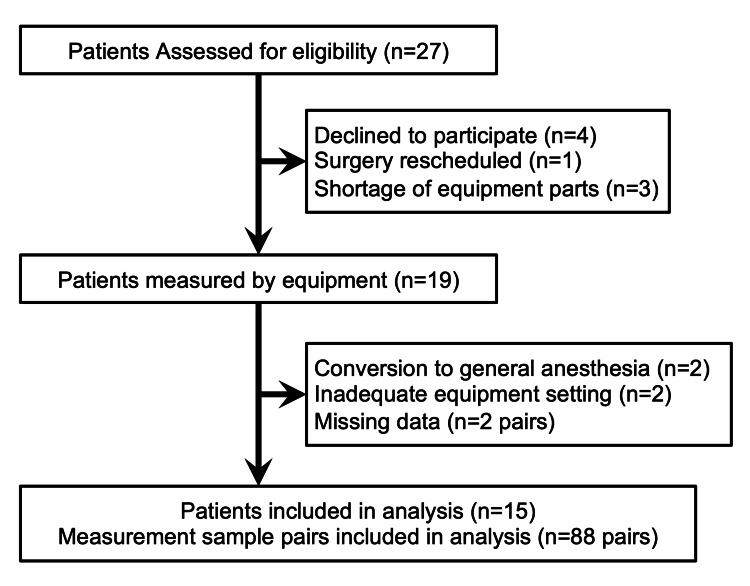
Study flow diagram.

We included 27 patients from November 1, 2020, to April 30, 2021. Of these, four patients were excluded because they declined to participate. One patient was excluded for a rescheduled surgery, and three patients were excluded because of a shortage of device components. The remaining 19 patients underwent measurements. However, two patients were converted to GA due to the need for transesophageal echocardiography for morphological assessment, two patients had inadequate device settings, and two pairs of results had missing measurements; therefore, they were excluded. Finally, we obtained 88 measurement pairs from 15 patients (Figure 1).

 The baseline participant characteristics are summarized in Table 1.

**Table 1:** Baseline characteristics of participants who underwent transaortic valve replacement. Data are presented as the mean (standard deviation) or absolute number (%). STS = Society of Thoracic Surgeons; eGFR = estimated glomerular filtration rate; TIA = transient ischemic attack; ABI = ankle-brachial index; LVEF = left ventricular ejection fraction; AVA = aortic valve area

Variable	Overall (n = 15)
Age (years)	84.73 (5.38)
Female patients	12 (80%)
STS score (%)	5.37 (2.24)
Height (cm)	149.55 (9.93)
Body weight (kg)	55.02 (9.85)
Hypertension	13 (87%)
Diabetes mellitus	4 (27%)
Dyslipidemia	7 (47%)
Atrial fibrillation	3 (20%)
Chronic kidney disease (eGFR < 60)	10 (67%)
Prior stroke/TIA	0 (0%)
Peripheral arterial disease	1 (7%)
Right ABI	1.09 (0.09)
Left ABI	1.08 (0.18)
LVEF (%)	63.87 (9.97)
AVA (cm^2^)	0.82 (0.28)
Peak pressure gradient (mmHg)	78.93 (21.05)
Mean pressure gradient (mmHg)	46.67 (14.09)

The anesthetic and surgical characteristics are summarized in Table 2.

**Table 2:** Anesthetic and surgical characteristics of participants who underwent transaortic valve replacement. Data are presented as the mean (standard deviation) or absolute number (%).

Variable	Overall (n = 15)
Anesthesia time (minute)	150 (20.56)
Procedure time (minute)	102.47 (18.22)
Balloon-expandable valve	13 (87%)
Self-expanding valve	2 (13%)
Norepinephrine therapy	1 (7%)
Total dose of norepinephrine (µg/kg)	0.65
Phenylephrine therapy	10 (67%)
Total dose of phenylephrine (µg/kg)	7.18 (7.45)
Ephedrine therapy	4 (27%)
Total ephedrine (mg/kg)	0.13 (0.07)
Nicardipine therapy	9 (60%)
Total nicardipine (µg/kg)	7.51 (5.28)
Dexmedetomidine	15 (100%)
Total dose of dexmedetomidine (µg/kg)	1.52 (0.30)
Propofol	14 (93%)
Total dose of propofol (mg/kg)	0.73 (0.35)
Midazolam	3 (20%)
Total dose of midazolam (mg/kg)	0.02 (0.01)
Fentanyl	15 (100%)
Total dose of fentanyl (µg/kg)	1.63 (0.33)

Primary outcome

**Figure 2:**
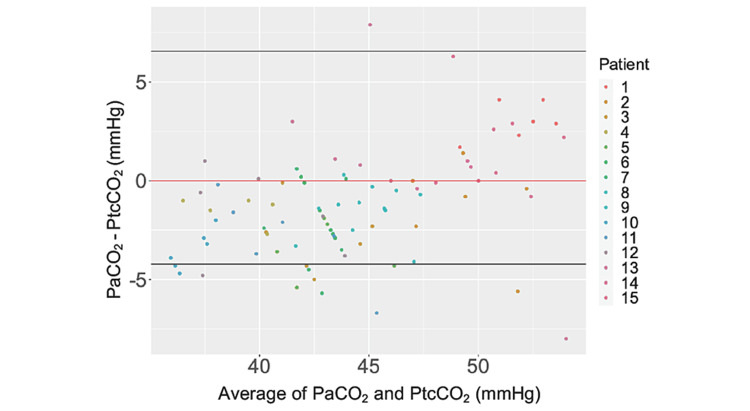
Bland–Altman plots for each set of pairs. The plot depicts the arterial carbon dioxide tension (PaCO_2_) value minus the transcutaneous carbon dioxide tension (PtcCO_2_) value (Y-axis) against the average of PaCO_2_ and PtcCO_2_ (X-axis). The black horizontal lines represent the 95% limits of agreement. The red horizontal line indicates zero.

Figure 2 presents the PtcCO_2 _and PaCO_2_ measurement results. Bland-Altman analysis indicated that the lower and upper 95% limits of agreement between the two measurements were -4.22 mmHg and 6.56 mmHg, respectively.

Before and after transcatheter aortic valve replacement

**Figure 3:**
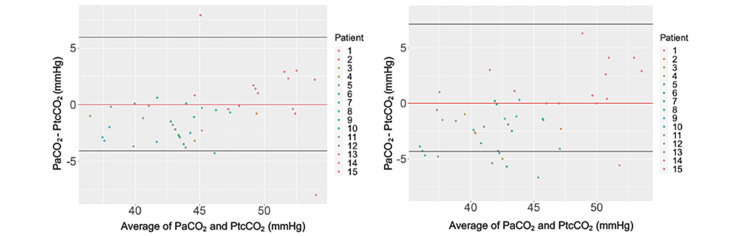
Bland–Altman plots for each set of pairs before (A) and after (B) transcatheter aortic valve replacement (TAVR). The plot depicts the arterial carbon dioxide tension (PaCO_2_) value minus the transcutaneous carbon dioxide tension (PtcCO_2_) value (Y-axis) against the average of PaCO_2_ and PtcCO_2_ (X-axis). The black horizontal lines represent the 95% limits of agreement. The red horizontal line indicates zero.

Figure 3 presents the measurements before and after valve implantation. The lower and upper 95% limits of agreement before valve implantation were -4.10 mmHg and 5.97 mmHg, respectively. The lower and upper 95% limits of agreement after valve implantation were -4.34 mmHg and 7.12 mmHg, respectively.

Post hoc analysis

Linear univariate regression analysis yielded a slope of 0.226 (p < 0.001), and an intercept of -11.183 (p < 0.001).

## Discussion

This study investigated the agreement between PtcCO_2_ and PaCO_2_ measurements during TAVR under MAC. We found that the lower 95% limit of agreement between the two measurements was >6 mmHg, which we had originally assumed to be the clinically acceptable margin; the difference was greater after valve implantation than it was before the procedure. We performed a post hoc regression analysis and found fixed and proportional errors.

Our results indicated that during TAVR under MAC with local anesthesia, PtcCO_2_ measurement could not provide a viable alternative to PaCO_2_ measurement to reduce high PaCO_2_ events. Previous studies assessed the agreement between PtcCO_2_ and PaCO_2_ in various situations, such as during GA [12], one-lung ventilation [13], deep sedation [14], after cardiac surgery [15], and in the surgical ICU [11]. These studies reported that the 95% limits of agreement between the two methods were within ±6 mmHg. However, these studies did not include patients who experienced rapid changes in their cardiovascular function, which can occur during the TAVR procedure [17-19]. Our results suggested that PtcCO_2_ measurement may be an unsuitable alternative to PaCO_2_ measurement during TAVR. Additionally, the post hoc analysis showed positive proportional errors. Therefore, PtcCO_2_ measurement could serve as an alternative to PaCO_2_, considering the trend that the higher the average value of PtcCO_2_ and PaCO_2_, the greater the difference between the two measurements. Further research is necessary to ascertain whether PtcCO_2_ can serve as an alternative to PaCO_2_ by devising a conversion equation between the two measurements.

Theoretically, the underlying mechanism of PtcCO_2_ instability might be hemodynamic change. Previous studies have shown that PtcCO_2_ measurements are unreliable in patients with low cardiac output [28] or major cutaneous vasoconstriction, which causes extremities or whole body livedo reticularis [29]. There are two possible hemodynamic changes during the TAVR procedure: hemodynamic changes due to pacing during valve implantation and hemodynamic changes due to the release of aortic valve stenosis after valve implantation.

During TAVR, rapid right ventricular pacing is used to perform balloon aortic valvuloplasty before prosthetic valve implantation [30,31] or to deploy the prosthetic valve in the appropriate position [1]. Rapid right ventricular pacing aims to reduce cardiac output from the left ventricle, and the systolic pressure target is <60 mmHg [31]. In cases of balloon-expandable valves, rapid pacing is essential. Conversely, for self-expanding valves, controlled pacing may be employed, allowing for continuous cardiac output [32]. Accordingly, significant hemodynamic changes occur during TAVR and may lead to differences in PtcCO_2_ and PaCO_2_ readings. However, our study did not directly measure the hemodynamic changes occurring during valve implantation. To clarify this mechanism, it is necessary to compare PtcCO_2_ and PaCO_2_ during valve implantation when hemodynamic changes are pronounced.

In addition to these hemodynamic changes during valve replacement, peripheral perfusion changes after valve replacement might cause inconsistency between PtcCO_2_ and PaCO_2_ measurements. A previous observational study using catheterization hemodynamic analysis reported that the relief of AS by TAVR causes a significant elevation of blood pressure and aortic forward expansion wave power [17]. In addition, pressure waveform analysis in this study revealed a slight increase in the delay of the backward pressure wave. Peripheral vasodilation following TAVR was suggested to have affected this delay [17]. Thus, TAVR may alter peripheral perfusion, which, in turn, may have affected PtcCO_2_ measurements. However, we did not directly evaluate peripheral perfusion in this study. To understand this mechanism, further research is needed to measure peripheral perfusion by non-invasive methods [33] and investigate TAVR-induced peripheral perfusion changes.

This study had several limitations. First, we did not evaluate the effects of vasoactive drugs on PtcCO_2_ measurements. However, in critically ill adult patients, the PtcCO_2_ and PaCO_2_ measurement biases were not different from patients receiving norepinephrine [11]. Therefore, we suspect that vasoactive drugs did not affect the PtcCO_2_ measurements. To clarify this point, future research is needed on the effects of vasoactive drugs on peripheral circulation in the earlobe. Second, we did not investigate the effects of sedative or analgesic drug administration timings or respiratory patterns during the procedure. Any change in respiratory status immediately before the PtcCO_2_ measurement could have affected the absolute numerical value of PtcCO_2_. It has been reported that PtcCO_2_ values tend to lag several minutes behind PaCO_2_ measurements in their response [22]. Thus, PtcCO_2_ values may not have immediately reflected changes in PaCO_2_ values associated with changes in respiratory patterns. Considering this influence, we planned to perform the measurements at times when the respiratory status was relatively stable. Third, we did not investigate the influence of various interventions, such as different types of artificial valves or balloon dilation, which can alter hemodynamics, on the timing of PtcCO_2_ measurements. In addition, we did not assess the variability in post-valve implantation hemodynamic recovery among cases. Future studies should examine how these changes in hemodynamics affect PtcCO_2_ measurements. Fourth, there were inadequate device settings and missing data. The inadequate device settings include issues such as insufficient adhesion of the sensor, the intrusion of air bubbles between the sensor and the earlobe, and damage to the equipment [14]. However, the required pre-determined sample size was achieved and, thus, these events had a minimal effect on the results.

## Conclusions

During TAVR under MAC with local anesthesia, PtcCO_2_ measurement could not provide a viable alternative to PaCO_2_ measurement to reduce high PaCO_2_ events. This study primarily focused on comparing the periods before and after valve implantation during TAVR. However, it is important to recognize that other factors, such as the type of prosthetic valve and the hemodynamic effects of balloon aortic valvuloplasty, can also significantly influence the procedure. Therefore, further investigation with increased sample size is warranted to understand how these different hemodynamic changes might impact PtcCO_2_ measurement during TAVR.
